# Strategies to mimic virally induced asthmatic exacerbations: the use of rhinovirus and influenza in acute and chronic mouse models

**DOI:** 10.1186/1476-9255-10-S1-P19

**Published:** 2013-08-14

**Authors:** RJ Shaw, M Sowa, MS Pedrick, M Fife, AT Nials, RG Knowles, E Hessel

**Affiliations:** 1 GlaxoSmithKline, Stevenage, Herts, England SG5 3QS, UK; 2 Arachos Pharma, Stevenage, Herts, England SG5 3QS, UK

## Rationale

Respiratory viruses are key in the development of exacerbations in COPD and asthma. We sought to characterise respiratory viruses to develop a model in which airway exposure to virus in addition to chronic allergen resulted in an exacerbated inflammatory and altered functional phenotype.

## Methods

BALB/c mice or human ICAM-1 transgenic mice (BALB/c background) were used in all assays. Animals were intranasally inoculated with either human rhinovirus 1B (HRV1B), human rhinovirus 16 (HRV16) or influenza A/Victoria/3/75 (H3N2). The kinetics of viral replication in naive animals and the lung inflammatory profiles in bronchoalveolar lavage (BAL) were assessed for up to seven days after single inoculations. The effects of HRV1B and H3N2 inoculation were further assessed in a model of chronic lung inflammation: animals were intranasally challenged with house dust mite extract (HDM) for up to seven weeks. Virus challenges were given after a robust HDM allergic phenotype developed. Some groups of animals were also treated with fluticasone propionate during and after the viral inoculation. After cessation of the challenge period, airway hyperresponsiveness (AHR) was assessed using whole body plethysmography, followed by BAL, serum and lung tissue analysis. In addition, the arterial oxygen saturation (PO_2_) of H3N2 treated animals was tested using pulse oximetry.

## Results

*In vitro* cytopathic effect assays did not demonstrate that either HRV1B or HRV16 were able to replicate in lungs of relevant mice. After viral inoculation, a variable inflammatory response was detectable only in HRV1B treated animals up to 24 hours post inoculation. Animals treated with H3N2 developed a mixed inflammatory lung inflammation and a PO_2_ profile indicative of a viral effect on lung functionality. H3N2 also replicated well in the mouse. Single inoculations of either HRV1B or H3N2 were given to animals already challenged with HDM. There was no indication that addition of HRV1B to existing phenotype altered the HDM induced AHR or inflammatory profile. Combination H3N2/ HDM treatment resulted in an increase in BAL eosinophils and neutrophils, but no change to steroid responsiveness nor changes in PO_2_ readouts.

## Conclusions

Strains of human rhinovirus are variably able to elicit lung inflammation but not AHR changes following single exposure to mouse hosts. H3N2 infection resulted in viral replication and a strong inflammatory response, cleared in 7 days following infection. We were unable to demonstrate that rhinovirus infection resulted in an exacerbation type response when given to allergic animals, but steroid sensitive inflammatory exacerbation was noted in HDM/ influenza treated animals.

